# Cacao in the Mississippian World: Archaeogenomic evidence for *T. cacao* consumption at the Etowah Site, Georgia

**DOI:** 10.1371/journal.pone.0353607

**Published:** 2026-08-26

**Authors:** Adam King, Terry G. Powis, Xavier Argout, Hélène Vignes, Jose Utge, Bénédicte Rhoné, Olivier Fouet, Nilesh W. Gaikwad, Claire Lanaud

**Affiliations:** 1 University of South Carolina, Columbia, South Carolina, United States of America; 2 Kennesaw State University, Department of Geography and Anthropology, Kennesaw, Georgia, United States of America; 3 CIRAD, AGAP Institut, Montpellier, France; 4 AGAP Institut, Université de Montpellier, CIRAD, INRAE, Institut Agro, Montpellier, France; 5 UMR 7206 Eco-anthropologie, Département Homme et Environnement, MNHN-CNRS-Université Paris Cité, Paris, France; 6 Gaikwad Steroidomics, LLC., Davis, California, United States of America; Institute for Biological Research, University of Belgrade, SERBIA

## Abstract

The discovery that people living at the great Ancestral Pueblo site of Chaco Canyon consumed cacao changed our understanding of the history of chocolate by demonstrating that networks moving cacao reached beyond Central and South America north at least into the American Southwest. Until now, clear evidence that people of the equally impressive Mississippian civilization east of the Mississippi River consumed chocolate has been lacking. This study presents the first evidence that cacao consumption was part of the Mississippian world at roughly the same time as it appeared at Chaco. We present evidence that samples from two Etowah Complicated Stamped pottery sherds recovered from 11^th^ to 12^th^ century contexts at the Etowah site in the modern state of Georgia contained highly fragmented ancient *Theobroma cacao* DNA. Etowah is an Indigenous city located in the modern US state of Georgia that, at its peak in the 14^th^ century, was one of the most important centers in the Mississippian world. Two centuries before that peak, Etowah’s inhabitants began construction of the site’s first earthen platform mound, excavating earth and filling the borrow pits with feasting remains. Our evidence for cacao consumption comes from these early feasting contexts. This discovery at Etowah asks us to broaden our understanding of the networks that distributed cacao throughout the Americas before European colonization.

## Introduction

For decades archaeologists, historians, and epigraphers have known that there is a long history of cacao (*Theobroma cacao*) consumption in Central America starting long before European contact. More recently it was evidenced that cacao consumption started at least 5,000 years ago in South America [1,2]. Clear evidence that Indigenous people north of modern-day Mexico also consumed cacao before the coming of Europeans has been much more difficult to find. Cacao is a plant that evolved and is adapted only to tropical climates, so its presence in precontact contexts in the modern-day US must have come from contact with people in or near cacao-producing areas like Central and South America. Using chemical residues absorbed into ancient pottery containers, Crown and Hurst [3] argued that Indigenous people living in Pueblo Bonito at the Ancestral Pueblo site of Chaco Canyon, New Mexico (1050–1350 CE) also consumed cacao-based drinks. They based this on the presence of what has been considered the key diagnostic compound of cacao, theobromine. Their argument was bolstered by additional lines of material evidence of sustained connections between the inhabitants of Chaco Canyon and people in tropical regions of modern-day Mexico [3].

**Fig 1:**
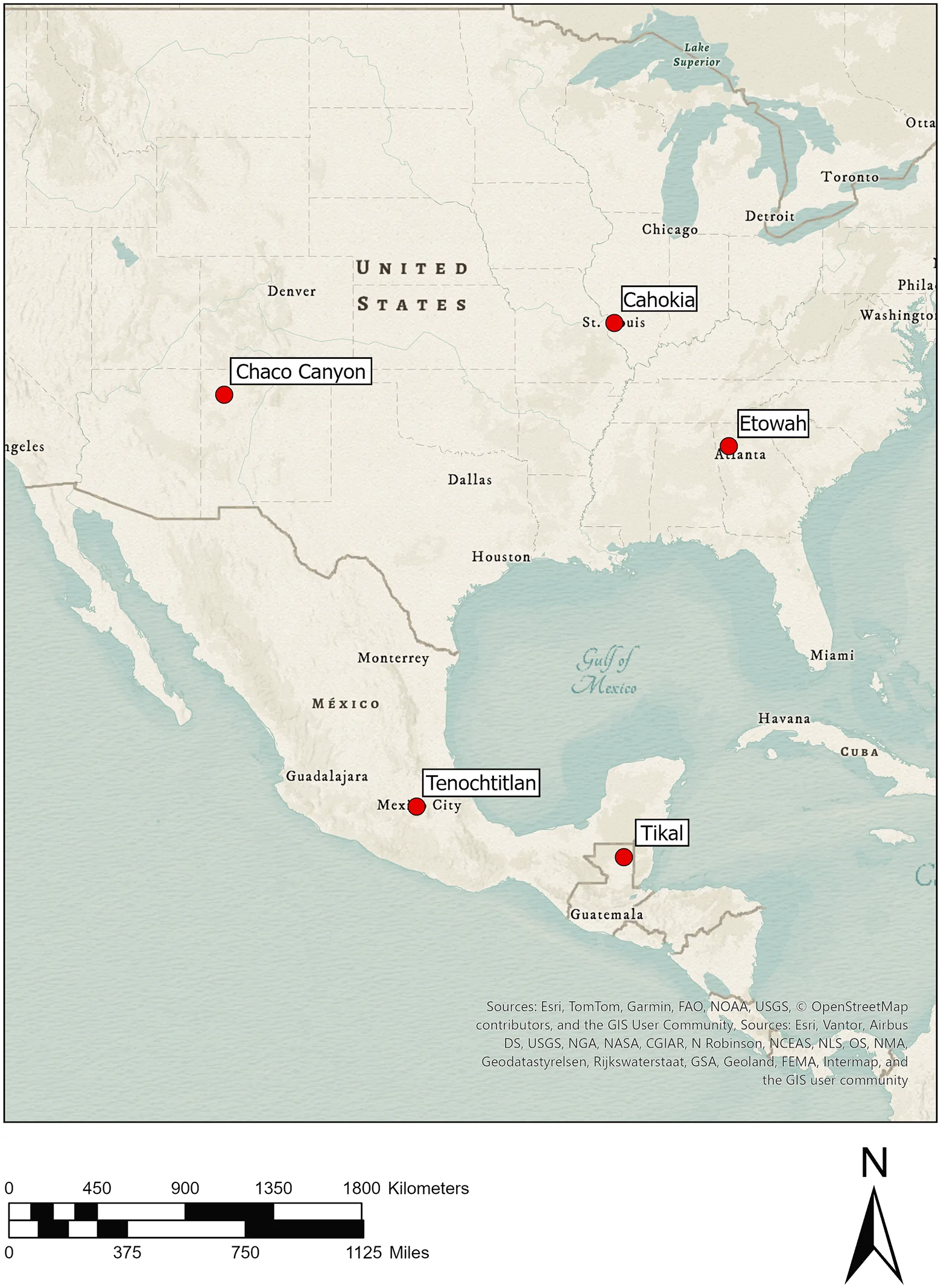
Location of Archaeological Sites and Regions Mentioned in the Text. Map data from OpenStreetMap (https://osmfoundation.org/wiki/Licence/Attribution_Guidelines).

Following this study, King et al. [4] attempted to use absorbed residues from Mississippian period (1050–1600 CE) pottery to search for evidence of cacao consumption in contemporary complex social and political forms in the Eastern US. While there is clear material evidence for connections between people living in Chaco Canyon and tropical regions of Central America where cacao grows, the same is not true of people living in the Eastern Woodlands of the US. Despite this, given the comparable scale and complexity of Mississippian period polities to the one centered in Chaco Canyon, we deemed this possibility worthy of exploration. To attempt this, King et al. [4] analyzed samples removed from pottery sherds recovered in 11^th^ to 12^th^ century feasting pits at the Etowah site (9Br1), a large Mississippian period (1050–1600 CE) city located in the modern state of Georgia (Fig 1).

Building on previous studies, the authors used mass spectrometry to detect four compounds known to be present in cacao: theobromine, theophylline, caffeine, and paraxanthine. Almost half of the Etowah samples contained at least two of the four diagnostic compounds. As part of that study, the authors also analyzed samples from cacao beans and yaupon holly (*Ilex vomitoria*), a plant that is native to the Gulf and Atlantic coasts of the US. The drink made from yaupon holly, called Black Drink by early European observers, is described extensively in historical accounts, has been found in archaeological contexts, and continues to be used today in ritual practice [5,6]. Contrary to Edwards and Bennett [7], King et al.’s [4] results show that in addition to theobromine and caffeine, yaupon holly also contains theophylline, once thought to be exclusively found in cacao. This led the authors to conclude, contrary to previous studies [8,9], it is not possible to distinguish ancient residues created by cacao consumption from those due to yaupon holly consumption using the commonly accepted biomarkers (caffeine, theobromine, and theophylline). King et al. [4] ultimately chose parsimony, arguing the residues detected at Etowah were most likely the result of yaupon holly consumption.

The study presented here uses ancient DNA analyses to move beyond the problems presented by the current state of absorbed residue studies. The analysis of ancient DNA makes it possible to directly observe the presence of useful plants in ceramic residues dating back several millennia, as was first demonstrated by [10] on amphoras. Although ancient DNA is characterized by high levels of degradation [11], specific cacao ancient DNA sequences have been identified in ceramic residues collected in the south of the Amazon in Ecuador [1] as well as from a wide range of ceramics belonging to 19 widespread cultures in South and Central America some of which dating back more than 5,000 years [2].

In this work we carried out ancient DNA analyses on three archaeological samples from Etowah, dating back 900–800 years, whose absorbed residues contained the three key compounds present in both cacao and yaupon holly. Archaeologists recovered the sherds from large feasting pits used as a source of fill to build the site’s first platform mound. Knight [12] has argued Mississippian period mound building was a communal right of intensification designed to renew the world. Our archaeogenomic results, especially when viewed in light of King et al.’s [4] absorbed residue results, argue strongly that cacao consumption was a practice known to the Mississippian world and, at least at Etowah, was part of a communal rites of intensification like mound building.

## Results

### Methylxanthine analyses

King et al. [4] first attempted to explore the possibility that people occupying Etowah during the Mississippian period consumed cacao-based drinks by conducting an absorbed residue study focused on samples collected from 31 pottery sherds recovered in the 1950s at the Etowah site. The sherds were recovered from a series of large features containing feasting remains associated with platform mound construction during the 11^th^ to 12^th^ centuries CE [13].

King et al. [4] collected samples from the 31 pottery sherds using a burr method outlined in [14] and analyzed them using a targeted mass spectrometry analytical approach (Table 1). Those authors used Evershed’s [15] biomarker method focusing on secondary metabolites of caffeinated drinks of the Americas, including caffeine, theobromine, theophylline, and paraxanthine. In the study, mass spectrometry detected all four alkaloids in only three samples (Samples 25, 3, 2 in Table 1), although 21 contained at least two of the four alkaloids (N = 31). After taking into account modern contamination, the authors concluded the majority of sherds sampled produced evidence of the consumption of caffeinated beverages. Because the same alkaloids are present in both cacao and yaupon holly, King et al. [4] concluded the most likely explanation was that locally available yaupon holly was the source of those residues rather than cacao.

**Table 1:** Xanthines detected in Etowah samples reported in King et al. 2017 [4].

Etowah	Theobromine	Theophylline	Paraxanthine	Caffeine
Samples	ng/gm	ng/gm	ng/gm	ng/gm
Sample 25	10.48	2.10	7.51	187.15
Sample 2	6.24	0.30	1.35	111.73
Sample 3	7.98	0.31	1.62	86.71
Sample 31	50.33			9.50
Sample 7		0.75	0.12	24.72
Sample 17		0.37	0.55	18.74
Sample 22		1.50	0.27	31.31
Sample 24		0.29	0.07	11.43
Sample 27		0.49	0.46	22.07
Sample 1		0.46		49.98
Sample 8		0.10		8.77
Sample 21		0.27		18.00
Sample 23		0.09		7.57
Sample 18		0.43		8.27
Sample 5		1.50		15.09
Sample 12			0.08	12.69
Sample 14			0.08	18.16
Sample 19			0.29	21.09
Sample 9			0.20	10.33
Sample 10			0.10	31.41
Sample 26			0.09	8.02
Sample 29				5.46
Sample 30				3.52
Sample 4				33.97
Sample 28				0.87
Sample 6				19.71
Sample 20				15.62
Sample 15				39.20
Sample 16				14.50
Sample 11				11.86
Sample 13				3.53

### Ancient DNA analyses

In the current study, the three samples containing all four key alkaloids present in cacao were analyzed for *T. cacao* ancient DNA. As noted above, ancient DNA is highly degraded, and is characterized by scarcity, postmortem deamination and decay. As a result, only small size fragments can be recovered. Following Lanaud et al. [2], we adapted the experimental conditions to avoid contamination in the first steps of experimentation (ancient DNA extraction, library constructions, see material and methods, Laboratory environment).

#### • Identification of *T.* *cacao* and wild relative species (Theobroma and *Herrania* genus) specific sequences.

502.9 million useful pair sequences (with quality scores and allowing to construct a consensus sequences) were produced after library construction and sequencing using targeted capture or whole genome sequencing (S1 Table).

**Fig 2:**
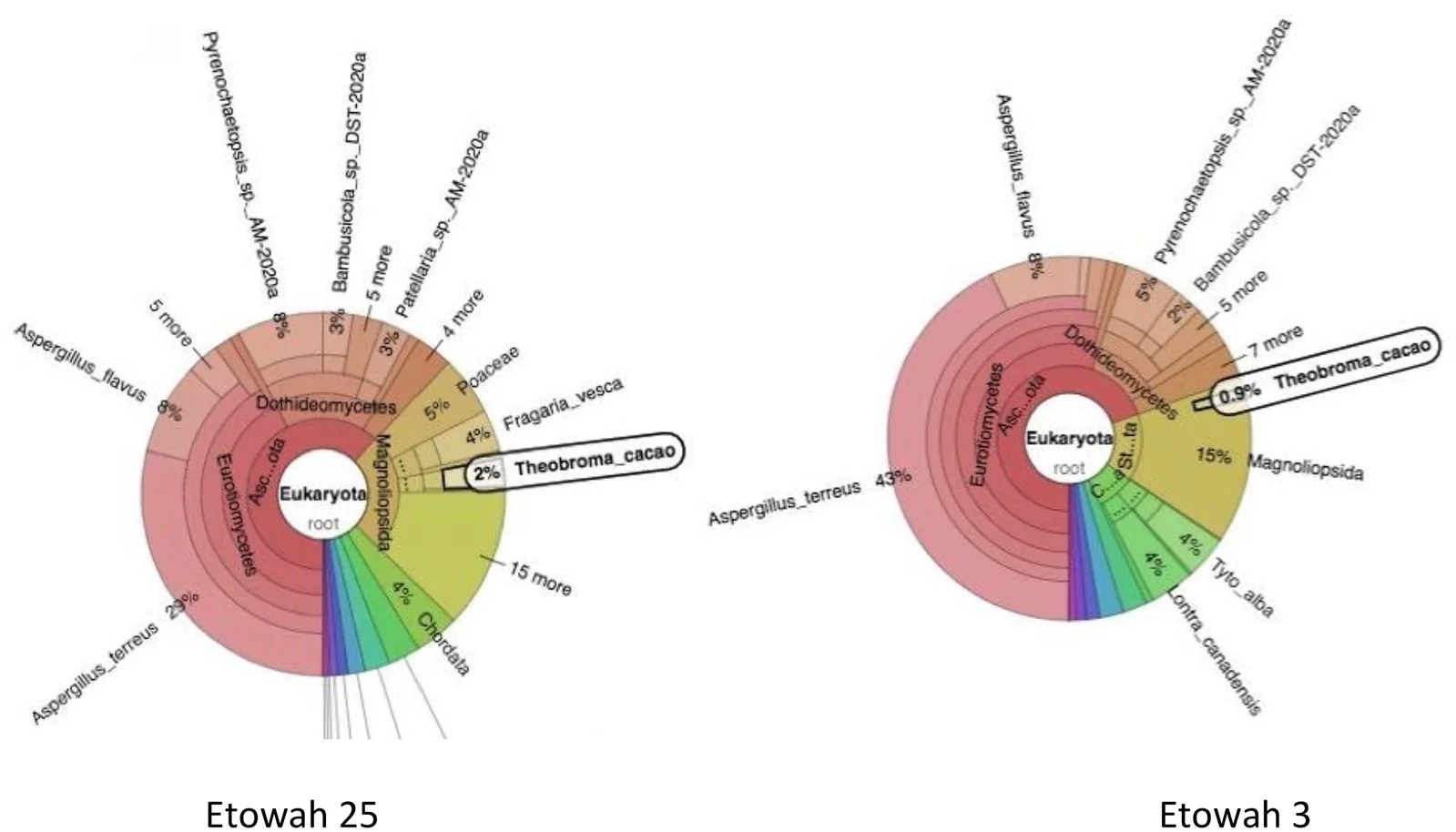
Visualization of metagenomic classification of ceramic residues sequences after mapping on the *T. cacao* genome. The Krona schemes [16] allow the visualization of relative abundances of taxonomical groups, identified as “first hit” after BLAST of the sequences on the Nr/Nt-NCBI international database among meta-genomic data obtained after mapping of whole sequencing data on the *T. cacao* genome, for two archaeological samples. The percentage of sequences identified as “first hit” *T. cacao* are visualized in the Figure.

The burr samples collected from the ceramics contained a mixture of DNA from several organisms. As many similarities are often observed between homologous sequences of the different species, two successive filters were applied to increase confidence of the presence of *T. cacao* sequences in the extracted ancient DNA:

- The first filter is the mapping of the ancient DNA sequences on the *T. cacao* genome. A total of 6360 sequences could first be mapped on the *T. cacao* genome (0.0012%). This set of sequences includes sequences from different species, including those of the *T. cacao* genome, homologous to the *T. cacao* genome sequences and selected with the standard genome mapping parameters.
- The second filter is a BLAST against the international database: NCBI Nr/Nt nucleotide collection. In Total, 36 sequences were then selected as specific *T. cacao* sequences, displaying the sequences as “first hit cacao” (Fig 2, S3 Fig). Only Samples 3 and 25, with respectively 12 and 18 specific *T. cacao* selected sequences, displayed at least 5 unique *T. cacao* specific sequences, considered previously [2] as the threshold to be used to affirm the presence of *T. cacao* in archaeological residue samples.

We hypothesized that none of the sequences selected were from closely related wild species or the result of contamination. This is because none of the sequences appeared as a first hit with wild relative species, and none of specific *T. cacao* sequences above this threshold were observed in the negative controls.

#### • Typical ancient DNA signatures.

When working with ancient DNA, post-mortem depurination [17,18] often leads to DNA strand fragmentation, which in turn leads to high postmortem ancient DNA fragmentation damages and a majority of small size fragments is observed. We observed these typical ancient DNA signatures in our Etowah residues analyses through two different approaches:

1) Fragment size distribution: MapDamage V2.2.1. [19] shows the fragment size distribution and only small size fragments (mean length of 84 bp) can be observed in the Etowah samples, as shown in S1 Fig.
2) Impact of amplified ancient DNA fragment size on PCR intensity:

When increasing the length of amplified DNA fragments, it is characteristic of ancient DNA for the intensity of the PCR (polymerase chain reaction) to be decreased, meaning that longer fragments failed to be amplified or are poorly amplified compared to smaller fragments. By evaluating the relative fluorescence units (RFUs) plotted against cycles number, we observed a decreased PCR amplification intensity or no amplification observed with primers amplifying the longer ancient DNA fragment (543 bp). This was not the case for the two modern T. cacao positive controls: Criollo, and Amelonado (S2 Fig) for which longer and smaller DNA fragments are amplified with a similar intensity.

#### • Preliminary Analysis of the Ancestry of *T. cacao* ancient DNA.

As [2] had done, we tried to elucidate the ancestry of the cacao traces found in Etowah’s Samples 3 and 25 by comparing them with a modern reference collection that includes representatives of the 11 *T. cacao* genetic groups [20] and 5 individuals belonging to 4 wild relative species and taken as a unique group (S2 Table).

After SNP (Single Nucleotide Polymorphisms) extraction within the *T. cacao* specific sequences of Sample 3 and Sample 25, respectively 20 and 18 SNP were identified, common to the reference collection and each of the archaeological samples. They allowed us to evaluate the Nei genetic distance between each archaeological sample, individually analyzed, and each genetic group (Table 2).

**Table 2:** Nei genetic distances between Etowah ancient DNA and modern DNA from *T. cacao* and wild relative genetic groups.

Etowah Archaeological Samples		Sample 3	Sample 25
Extraction name		MX11-MX18	MX10
Number of SNP		20	18
Genetic groups	Criollo	0.223	0.325
	Caqueta	0.231	0.225
	Curaray	0.223	0.059
	Nacional	0.207	0.118
	Purus	0.294	0.217
	Contamana	0.249	0.220
	Marañon	0.116	0.097
	Iquitos	0.181	0.289
	Amelonado	0.052	0.294
	Nanay	0.262	0.340
	Guiana	0.261	0.305
	Wild relative sp. group	0.762	0.572

Genetic distances were calculated for each archaeological sample with the GENETIX software V4.05.2 [21], and the Nei 78 distances [22] adapted to small effective size. Twelve genetic groups were considered: the eleven genetic groups recently reported^4^ and a wild relative group including *T. bicolor*, *T. grandiflorum*, *T. speciosum* and *Herrania nitida*.

Based on the SNP similarities between *T. cacao* specific sequences identified in Samples 3 and 25, and homologous sequences from modern *T. cacao* genetic groups and wild species accessions, we observed that a closer genetic distance between Sample 3 and the Amelonado genetic group, followed by the Marañon group. For Sample 25, its closer genetic distance was observed with the Curaray group, followed by the Marañon group. Given the low number of SNP identified in the cacao specific sequences, these finding represent preliminary results of ancestry that must be confirmed by the identification of new specific cacao sequences and subsequent SNPs.

## Discussion

There is very little material evidence of connections between people living in Mississippian places like Etowah and those living in Central America. Similarly, there is very little direct evidence that the inhabitants of Ancestral Pueblo or Hohokam sites of the American Southwest maintained sustained contacts with people in the Eastern Woodlands. As a result, the possibility that the Mississippian world had access to cacao has always seemed remote. The methods used to identify ancient chemical residues of cacao cannot distinguish between it and a drink containing yaupon holly, making it difficult to explore the possibility of finding cacao in the precontact Eastern Woodlands [4]. Archaeogenomics has the potential to change that.

In this study, we searched for specific *T. cacao* ancient DNA sequences in samples recovered from pottery sherds found at the Etowah site. Ancient DNA is very degraded, and during PCR steps, a potential contamination of samples by modern cacao DNA could lead to preferential amplification of this cacao modern DNA, generating false results. For these reasons, we took the maximum precautions to avoid the contamination of the archaeological samples with modern cacao before and during all the steps of ancient DNA analyses. As mentioned in Material and Method section, all samples were stored in bags within boxes from their excavation until their analysis. Then, ancient DNA extraction and library construction were conducted under sterile conditions in a platform dedicated to ancient DNA analyses and equipped with positive high-pressure air system. After sequence analyses, we looked for typical ancient DNA signatures to ensure that modern cacao DNA has not contaminated the samples. Indeed, as ancient DNA is very degraded, a small size DNA fragments distribution is generally observed. Such small size DNA fragments distribution was revealed by two methods: by direct sequencing and by a decreased PCR amplification intensity using primer pairs amplifying longer DNA fragments, compared to control modern DNA amplification.

No remains of cacao seeds or other preserved portions of cacao plants have been recovered from Etowah, so only samples collected from the walls of ceramic containers were available to detect the presence of *T. cacao*. Those ancient food residue samples contain a mixture of DNA from different species belonging to the environment, including plants, bacteria, and animals. So, in addition to strict experimental conditions aiming to avoid external *T. cacao* contamination, we applied two successive stringent bioinformatic filters to ensure the presence of *T. cacao* specific ssequences and discarding any sequences common to several other species that could lead to ambiguous *T. cacao* identification. Because the sherds sampled had been washed previously, only a relatively small number of *T. cacao* sequences were recovered that could be analyzed. Despite this, our results revealed the presence of specific *T. cacao* ancient DNA sequences in the samples collected from two of the three Etowah sherds analyzed.

It is important to recall that King et al.’s [4] absorbed residue study found alkaloids diagnostic *T. cacao* (see Table 1) in Sample 3 and Sample 25. While we acknowledge that two vessels is a small number of results, it also is important to note that 21 of the samples analysed in that absorbed residue study contained at least two of those diagnostic methylxanthine alkaloids (N = 31). Given the correspondence between the absorbed methylxanthine residues and genomic results for Sample 3 and Sample 25, we are confident that the other absorbed residue samples containing diagnostic *T. cacao* methylxanthines also could result from the presence of *T. cacao*. As a result, despite the fact that Etowah is separated from potential sources of cacao by great distances, ancient DNA confirms the site’s inhabitants consumed cacao.

Finding evidence for the consumption of cacao at Etowah begs the question of its source and the nature of the networks that connected the Mississippian world to cacao-growing regions. Unfortunately, that is difficult to determine using the ancient DNA results because, as Lanaud et al. [2] demonstrated, genetic groups that originated in different regions of South America also were introduced into Central America in the deep past. Moreover, our findings rely on a small number of SNP. However, our results show the Sample 3 sequence is most closely related to the Amelonado and Marañon genetic groups, while Sample 25’s sequence is closest to the Curaray and Marañon groups. Cacao deriving from these genetic groups has been identified in ancient Maya contexts in Belize (600BCE–250CE), as well as in Jama Coaque contexts in Ecuador (350BCE-1532CE) [2].

Given this, it is possible that cacao came to Etowah from regions in Central America, closer to Georgia than Ecuador, via networks that moved things, people, and ideas. It was such a network that brought other materials from tropical Central America such as copper bells, cloisonne’, and Scarlet Macaws to Chaco Canyon [3]. While a different network may have brought cacao into the Eastern Woodlands through northern Mexico, it is also possible that people living in the American Southwest were the source of Etowah’s cacao. While it is worth noting that cacao consumption in Chaco Canyon is contemporary with the dating of Etowah’s ancient cacao DNA sequences, neither direct connections to Mexico nor the American Southwest are supported by archaeological evidence for now.

These results ask us to reopen lines of research long closed, especially regarding contacts between the impressive civilizations of Central and South America and the great Mississippian civilization of the Eastern Woodlands. Early in the history of Mississippian studies, it was common to attribute the complexity of Mississippian lifeways to connections with Central America. This line of explanation was eventually abandoned for lack of supporting evidence and because of the implication that Indigenous people of the Eastern Woodlands were not capable of creating a complex civilization. As new evidence comes to light, it will become time to explore those connections, but this time without denying the achievements of Mississippian people.

At the same time, these results ask us to look more closely for evidence of links connecting the Mississippian world to the Hohokam and Ancestral Pueblo of the Southwest. There is growing evidence of links between people of the Southwest and societies on the western edge of the Mississippian world known by archaeologists as Caddoan Mississippian centered on the modern-day states of Arkansas, Louisiana, Oklahoma, and Texas. Those connections are evidenced by the presence of small amounts of turquoise, Southwestern pottery styles, and obsidian that presumably made their way across the Great Plains to Caddoan Mississippian contexts from 800 to 1600 CE [23].

The Etowah pottery sampled for this study was found in large features dating to the 11^th^ to 12^th^ centuries CE [13]. These contexts are early in the history of Etowah, over a century before it became one of the most important centers in the Mississippian world [13]. This dating places cacao consumption at Etowah early in the Mississippian period, suggesting the need to explore the possibility that cacao was part of the beginnings of Mississippian civilization. Given this, it is possible that the absorbed residue studies finding theobromine, theophylline, and caffeine in vessels from the early Mississippian city of Cahokia [5], as well as in results published by Washburn et al. [8] from the Ohio and Mississippi River valleys actually reveal the presence of cacao instead of, or in addition to yaupon holly. Only additional archaeogenomic studies can investigate this hypothesis. More broadly, our results suggest that it is time to explore the possibility that cacao was distributed more widely in the precontact Americas than we have understood.

It is equally important to understand that archaeologists recovered the sherds sampled from large features that served as sources of fill for the first earthen platform mound at Etowah. Etowah’s inhabitants quickly filled those features with dense concentrations of animal bone, broken pottery, and organic materials that have been interpreted as the remains of feasting [24,13]. Using historical and linguistic evidence, Knight [12] argued that Mississippian period mound building was a world renewal ritual that, as a communal right of intensification, built social solidarity. Following this, King [25] and [26] have argued the construction of the first mound at Etowah was an important part of binding together the disparate social groups who first established Etowah. This suggests that, at least at Etowah and possibly across the Mississippian world, cacao consumption played a role in world renewal rites of intensification.

## Materials and methods

### Origin of archaeological items

The pottery sherds whose samples are analyzed for this paper were recovered from the Etowah Indian Mounds State Historic site (9Br1), located in Bartow County, Georgia (34°7′30.47″N 84°48′27.59″W). Arthur R. Kelly and students from the University of Georgia excavated them from a series of borrow pits filled with feasting remains from 1954 to 1957 [13]. The sherds fit the type Etowah Complicated Stamped and, although small, were portions of flaring rim jars. The features where Kelly and crew recovered them were located between Mounds A and B and date to the 11^th^ and 12^th^ centuries CE [13]. From the 1950s until 2013 when the samples were collected, the sherds were washed and stored in paper bags and shoeboxes then transferred to plastic bags. They currently are housed at the University of Georgia’s Laboratory of Archaeology in Athens, GA.

### Potential contamination

There are many places from recovery through analysis where the pottery sherds we analyzed could have been contaminated by modern chocolate. It is possible that archaeologists introduced chocolate to the pottery during excavation, during washing, and as it was transferred from one storage medium to another. Given the post-recovery history of the pottery sampled, we cannot be sure modern chocolate was not introduced at some point from initial recovery to final storage. As indicated in the Ancient DNA Analysis and Discussion sections of this paper, archaeogenomic analysts were able to confirm that the DNA recovered is from ancient sources and not the result of contamination with modern cacao.

Because the pottery sampled for this study was recovered from a feasting feature, it is possible that ancient cacao DNA was introduced to the sherds sampled after being deposited. Feasting features are complex archaeological contexts that can contain a wide variety of different things, among them various plant and animal remains. Under these circumstances, DNA and other residues not originally held by containers could be introduced after deposition. While we cannot be sure the pottery vessels we sampled actually held cacao, for our purposes the presence of ancient cacao DNA is the significant finding.

### Collection of ceramic residues

Samples from Etowah site pottery sherds were collected using a burr method developed by [14]. The interior surfaces of pottery sherds were abraded with clean sandpaper, the burr was collected on clean paper and transferred to clean plastic vials for storage and transport. During sample collection, King and Powis wore gloves and masks to reduce modern contamination.

### Methylxanthine analyses

The pottery sherd samples were analyzed at the University of California at Davis’ Department of Nutrition using ultra-performance liquid chromatography mass spectrometry (UPLC-MS/MS). Following previous studies, these analyses focused on the detection of theobromine, theophylline, caffeine, and paraxanthine. Burr samples were incubated with 200 ml milli-Q water at 80°C for 30 min. After incubation samples were vortexed and centrifuged. The resulting sediment from each sample was removed and the supernatant filtered using 5 kD membrane filters. Filtrates were transferred to vials for UPLC/MS-MS analysis. Modern pottery samples were extracted and included in the analysis as controls. UPLC/MS-MS analyses of all the samples were conducted using a Waters Acquity UPLC system connected with Xevo-TQ triple quadruple mass spectrometer. Analytical separations on the UPLC system were conducted using an Acquity UPLC C18 1.6 mm column (2, 150 mm) at a flow rate of 0.15 ml/min. The gradient started with 100% A (0.1% formic acid in H_2_O) and 0% B (0.1% formic acid in CH_3_CN), changed to 50% A over 5 min, followed by a 5-min linear gradient to 10% A, resulting in a total separation time of 10 min. The elutions from the UPLC column were introduced to the mass spectrometer.

Subsequently, a Xevo-TQ triple quadruple mass spectrometer (Waters, Milford, MA, USA) recorded MS and MS-MS spectra using Electro Spray Ionization (ESI) in positive ion (PI) mode, capillary voltage of 3.0 kV, extractor cone voltage of 3 V, sample cone voltage

of 32 V, and detector voltage of 500 V. Cone gas flow was set at 50 L/h and desolvation gas flow was maintained at 600 L/h. Source temperature and desolvation temperatures were set at 150 and 350°C, respectively. The acquisition range was 20e300 Da. Pure standards of caffeine, paraxanthine, theobromine and theophylline were introduced to the source at a flow rate of 10 ml/min by using methanol: water (1:1) and 0.1% formic acid mixture as the carrier solution to develop multiple reaction monitoring (MRM) method for UPLC/MS-MS operation. Resulting data from LCMS analysis from all samples were analyzed and processed using MassLynx 4.1 software.

### Laboratory environment for ancient DNA analyses

Ancient DNA is highly degraded and characterized by scarcity and damage due to post-mortem decay and deamination [11]. To prevent contamination by modern DNA, which is preferentially amplified during PCR steps, the experimental conditions were adapted: all pre-PCR experiments were conducted under sterile conditions in the platform “Paléogénomique et génétique moléculaire” (P2GM) of the French “Muséum National d’Histoire Naturelle” at the “Musée de l’Homme” (Paris). This laboratory is dedicated to ancient DNA analyses and equipped with positive high-pressure air system, with continuous filtering of incoming air, daily UV light irradiation, laminar flow hoods with HEPA filters, and all surfaces frequently cleaned. The experimenters wore whole-body protective clothing including gloves and shoe protection.

### Extraction of ancient DNA

Ancient DNA extraction was made from 0.5g of ceramic residue burr samples, following [2], with the Qiagen, DNeasy PowerLyzer PowerSoil Kit. This effectively removes PCR inhibitors such as humic acids, and according to the described manufacturer’s procedure except for the binding step for which the concentration of the C4 saline solution was increased 1.6 times to retain the smallest fragments. Negative controls were processed alongside the ceramic residue samples.

### Libraries construction and sequencing steps

Libraries construction and sequencing steps were performed according to Lanaud et al. [2]: Construction of the libraries using the NEXTflexTM Rapid DNA-Sequencing Kit (Bio Scientific) were carried out for MX10 and MX11 according to the protocol indicated in the kit. Dual-indexed Illumina sequencing libraries were prepared for MX17 to MX19 using three main reactions: blunt end-repair, adapter ligation, and nick fill-in reaction [27].

The ancient DNA libraries were sequenced on a NOVASEQ 6000 Illumina sequencer according to two possible different strategies reported for each sample in S1 Table or directly by whole genome sequencing (WGS) or after a step of targeted capture (TC) as described previously [1]. The pipeline used for filtering, demultiplexing, and processing the reads is already described [28].

### Bioinformatic sequences treatment

A mixture of several species of bacteria, fungi, plants, and animals can be present in the collected ceramic residues. Thus, two successive filters were necessary to specifically identify the *T. cacao* sequences. The first filter was the mapping against the *T. cacao* genome (V2) [29] and a second filter allowed us to identify the sequences specific to *T. cacao* through a BLAST (Basic Local Alignment Search Tool) against data sequences from the international database: NCBI Nr/Nt nucleotide collection. We used Krona schemes to visualize the relative abundances of the several sequences species within the metagenomic classifications and identified as “first hit” after blast against the NCBI NT database [16], as represented in Fig 2.

The successive bioinformatic steps needed to select specific *T. cacao* sequences were carried out according to Lanaud et al. [2].

### Ancient DNA authentication

Pre-selected ancient DNA fragments, obtained after aligning the reads on the *T. cacao* genome were processed. Small size fragments, resulting from postmortem depurination were evaluated with 2 different approaches:

1) the read lengths distribution was reported using MapDamage V2.2.1. [19] (S1 Fig)
2) Real-time PCR amplifications of ancient DNA extracts were carried out to check the relationship between PCR amplification intensity and length of amplified ancient DNA fragments (S2 Fig).

Primers pairs (S3 Table) corresponding to the amplification of increased lengths of mitochondrial “*Cytochrome C Oxidase* subunit 2” DNA fragments were designed to compare their relative efficacy for amplifying ancient DNA extracts: (red) Mito6: 100 bp; (blue) Mito 290: 290 bp; (purple) Mito 543: 543 bp. Two positive controls corresponding to modern DNA from Criollo (B97/61) and Amelonado (Catongo) varieties were included in the experiment.

Real-time PCR analysis was carried out in a BioRad CFX96 Touch Real-Time PCR Detection System (Bio-Rad Laboratoires) using the following steps: 98°C for 3 min; a touchdown PCR [initial 10 cycles; 98°C for 15 s, 58°C for 20s (−1°C every cycle), 72°C for 20s], followed by 50 cycles of 98°C for 15°C, 48°C for 20 s, 72°C for 20s, then a final step at 72°C for 8 min. To confirm product specificity a melting curve analysis was performed as the last step. The real-time PCR was carried out in a 10 μl reaction volume containing 5 μl of 2X « SsoFast EvaGreen Supermix » (Bio-Rad Laboratoires), 0.1 mM of BSA (ref. B9200, New England BioLabs), 500 nM of forward and reverse primers (S3 Table), 1 µl of water, and 2 µl of DNA extracts. In each run, a blank (negative control) and positive control were added.

Data were analyzed using CFX Maestro Software (Bio-Rad Laboratoires) set with default parameters to determine the cycle threshold (*C*T), *i.e.,* the number of PCR cycles required for the fluorescent signal to exceed the background level.

### SNP identification

SNP identifications were carried out in the specific *T. cacao* sequences identified in the three Etowah samples and as previously described [2] to identify the ancestry of Etowah archaeological residue samples compared to the eleven modern genetic groups of the *T. cacao* species recently identified [20] and to the wild relative species.

We used a re-sequencing and pan-genome project of 216 modern cacao accessions, recently carried out [28], which allowed to identify 31,910,149 SNPs. Among them, SNP from a subset of 76 cacao accessions representative of the genetic diversity of the species and five relative wild species were selected to perform genetic distance analyses, based on common sets of markers, between this reference collection and each archaeological item.

To take into account the potential deaminated DNA damage at the ends of the fragments, and avoid false SNP, we followed the suggestion to remove the SNP, corresponding to potential C-to-T/G-to-A substitutions, located at the two first bases of each end of ancient DNA fragments [30].

### Reference collection

A reference collection representing the 11 genetic groups of *T. cacao* species was selected as previously [2] to identify the ancestry of Etowah archaeological samples. It includes representative of Criollo (8 ind), Caqueta (8 ind), Curaray (8 ind), Nacional (8 ind), Purus (7 ind), Contamana (4 ind), Marañon (7 ind), Iquitos (7 ind), Amelonado (4 ind), Nanay (8 ind), Guiana (7 ind). This collection includes also five accessions from four wild relative species: *Herrania nitida* (2 ind)*, T. grandiflora* (1 ind)*, T. bicolor* (1 ind)*, and T. speciosum* (1 ind), considered as a unique group*.*

### Genetic distance analyses

The genetic distance is a measure of genetic differences/divergence between two populations or organisms. It measures the accumulated allele differences per locus. Based on SNP differences between ancient DNA sequences, selected as specific *T. cacao* sequences, and homologous modern cacao DNA sequences we calculated the Nei genetic distances [22], adapted to small effective size, between the ancient DNA extracted from Samples 3 and 25, and the other 12 modern genetic groups (including *T. cacao* groups and the five wild *Theobroma* and *Herrania* accessions taken as a separate group) using GENETIX software V4.05.2 [21] (Table 2).

## Supporting information

S1 Fig Single-end read length distribution of the three Etowah analyzed samples. The residues from the three archaeological samples Etowah 2, 3 and 25 display small size read lengths, revealed by MapDamage V2.2.1. [19], as generally observed in ancient DNA.
(PDF)

S2 Fig Evidence of high level of fragmentation in ancient DNA. Real-time PCR amplifications of aDNA extracts were carried out. Primers pairs corresponding to the amplification of increased lengths of mitochondrial “*Cytochrome C Oxidase* subunit 2” DNA fragments were used to compare their relative efficacy for amplifying aDNA extracts: (red) Mito100: 100 bp; (blue) Mito 290: 290 bp; (purple) Mito 543: 543 bp. The relative fluorescence units (RFUs) is plotted against cycles number. A decreased PCR amplification intensity or no amplification can be observed with primers amplifying the longer aDNA fragment (543 bp) in the three Etowah samples, which is not observed in the two modern *T. cacao* positive controls:, Amelonado and Criollo samples.
(JPG)

S3 Fig Ancient DNA sequences selected as specific *T. cacao* sequences. The sequences obtained after library sequencing on a NOVASEQ 6000 Illumina sequencer were selected as “First hit” *T. cacao* after mapping on the *T. cacao genome* followed by a BLAST made on the international database: NCBI Nr/Nt nucleotide collection.
(DOCX)

S1 Table Results of aDNA analyses from residues collected in the Etowah samples. Two sequencing strategies were used: TC: targeted capture and WGS: whole genome sequencing. After sequencing, two successive filters allowed to select only the sequences specific to *Theobroma cacao* (first hit *T. cacao*): a first mapping on the *T. cacao* genome, and a BLAST against the international database: NCBI Nr/Nt nucleotide collection.
(DOCX)

S2 Table Reference collection. Origin of modern accessions used to establish the Nei genetic distance of Etowah samples to the several *T. cacao* and wild relative genetic groups. This collection contains representatives of the eleven groups identified [20] (76 accessions) and 5 individuals from four wild relative species: three *Theobroma* species and one *Herrania* species. INTA: Nicaraguan Institute of Agricultural Technology (Nicaragua); CIC: Centro de Investigacion Caribia (Colombia); CRC: Cocoa Research Center (Trinidad and Tobago); CATIE: Centro Agronómico Tropical de Investigación y Enseñanza (Costa Rica); INIAP-EETP: Instituto Nacional de Investigaciones Agropecuarias (Ecuador); INIAP-ECCA: Instituto Nacional de Investigaciones Agropecuarias (Ecuador); CIRAD: Centre de Coopération Internationale en Recherche Agronomique pour le Développement (France).
(DOCX)

S3 Table Primer pairs used to show evidence of ancient DNA fragmentation. Three primers pairs defined in the mitochondrial *Cytochrome oxidase* gene and having a similar PCR efficiency were used to amplify DNA fragments differing for their size (100 bp, 290 bp, 543 bp). These primers allowed to check the decreased amplification level of ancient DNA with primers amplifying longer DNA fragments, which is a characteristic of ancient DNA.
(DOCX)
