# Supplementary material for: Cacao in the Mississippian World: Archaeogenomic evidence for T. cacao consumption at the Etowah Site, Georgia
Source: PLoS One. 2026 Aug 26;21(8):e0353607. doi: 10.1371/journal.pone.0353607 (PMC13514064; doi:10.1371/journal.pone.0353607)
Supplement: Supplementary file 1 [file pone.0353607.s001.pdf]

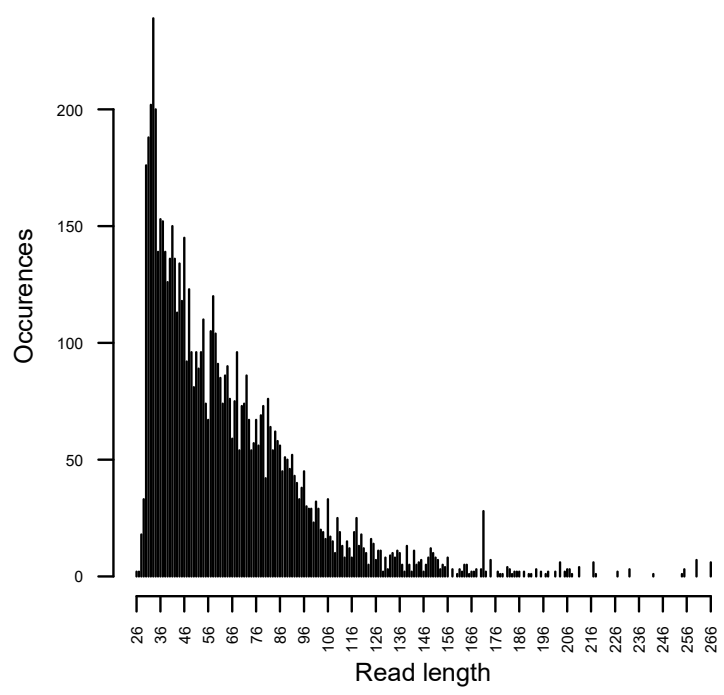

Etowah 2

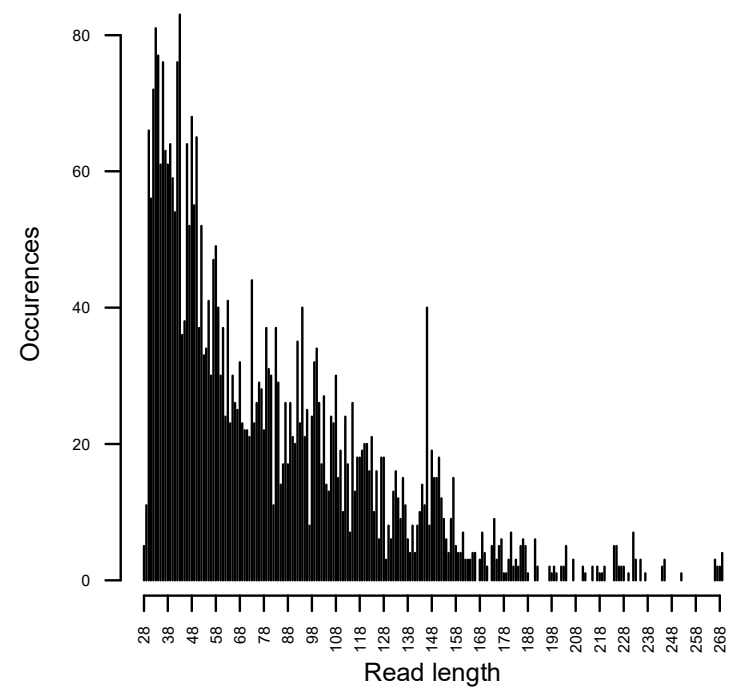

Etowah 3

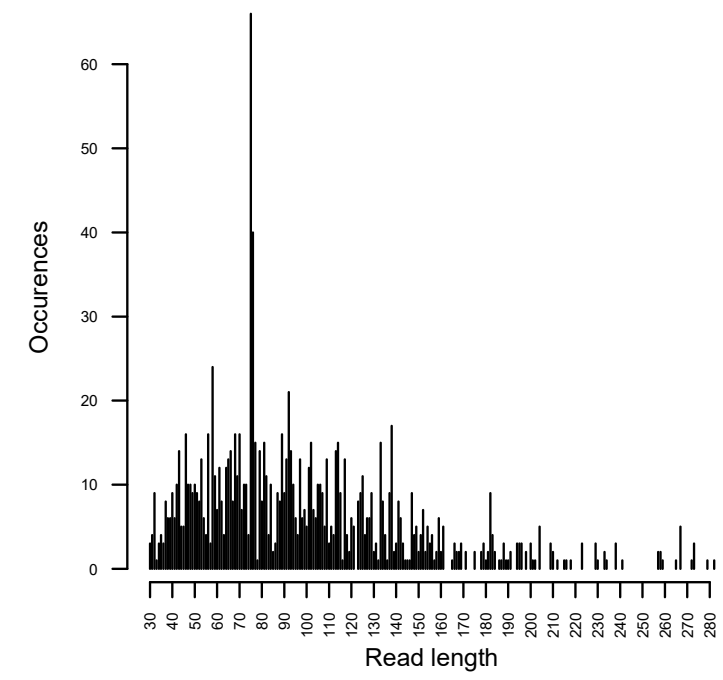

Etowah 25
