# Supplementary material for: Cacao in the Mississippian World: Archaeogenomic evidence for T. cacao consumption at the Etowah Site, Georgia
Source: PLoS One. 2026 Aug 26;21(8):e0353607. doi: 10.1371/journal.pone.0353607 (PMC13514064; doi:10.1371/journal.pone.0353607)
Supplement: Supplementary file 2 [file pone.0353607.s004.docx]

archaeological samples	DNA extraction name	Sequencing strategy	Total number of pair ends	useful pair numbers	number of unique sequences	unique sequences mapped on the cocoa genome	number of sequences 1st hit *T. cacao*
Etowah 2	MX17	TC	94141804	88540515	6786464	876	2
Etowah 2	MX17	WGS	83718301	75765459	7529796	2974	4
Etowah 3	MX11-MX18	TC	209282753	179421098	21982317	1364	11
Etowah 3	MX18	WGS	57669134	18839400	834093	177	1
Etowah 25	MX10-MX19	TC	150070997	140375150	32433013	969	18
Total			594882989	502941622	69565683	6360	36
