# Supplementary material for: Cacao in the Mississippian World: Archaeogenomic evidence for T. cacao consumption at the Etowah Site, Georgia
Source: PLoS One. 2026 Aug 26;21(8):e0353607. doi: 10.1371/journal.pone.0353607 (PMC13514064; doi:10.1371/journal.pone.0353607)
Supplement: Supplementary file 3 [file pone.0353607.s005.docx]

Species	Accession	Genetic group	Preservation collection	Country of origin	Place of collection
Theobroma cacao	B_97 61/B2	Criollo	CRC	Belize	Toledo district
Theobroma cacao	B97_EX1_C3	Criollo	CRC	Belize	Toledo district
Theobroma cacao	CACAO_2	Criollo	Cirad-DNA stock	México	Yucatan
Theobroma cacao	COL_10	Criollo	CIC	Colombia	Magdalena
Theobroma cacao	HABILLAL	Criollo	Cirad-DNA stock	México	Michoacan
Theobroma cacao	HE_4	Criollo	Cirad-DNA stock	Venezuela	Tachira
Theobroma cacao	LAN_7	Criollo	Cirad-DNA stock	México	Lacandona forest
Theobroma cacao	LIB_3	Criollo	INTA	NICARAGUA	La libertad de Chontalpa
Theobroma cacao	EBC_10_401	Caqueta	Cirad	Colombia	Rio Caqueta, Amazonas
Theobroma cacao	EBC_29	Caqueta	INIAP-ECCA	Colombia	Rio Caqueta, Amazonas
Theobroma cacao	EBC_30	Caqueta	INIAP-ECCA	Colombia	Rio Caqueta, Amazonas
Theobroma cacao	EBC_48	Caqueta	INIAP-ECCA	Colombia	Rio Caqueta, Amazonas
Theobroma cacao	EBC_5	Caqueta	Cirad	Colombia	Rio Caqueta, Amazonas
Theobroma cacao	EBC_6_401	Caqueta	Cirad	Colombia	Rio Caqueta, Amazonas
Theobroma cacao	EBC_6_S10	Caqueta	INIAP-EETP	Colombia	Rio Caqueta, Amazonas
Theobroma cacao	EBC_9_A2	Caqueta	INIAP-ECCA	Colombia	Rio Caqueta, Amazonas
Theobroma cacao	LCTEEN_218	Curaray	INIAP-EETP	Ecuador	Rio Villano, Para
Theobroma cacao	LCTEEN_227	Curaray	INIAP-EETP	Ecuador	Rio Villano, Para
Theobroma cacao	LCTEEN_312	Curaray	INIAP-EETP	Ecuador	Rio Upano
Theobroma cacao	LCTEEN_333	Curaray	INIAP-EETP	Ecuador	Rio Upano
Theobroma cacao	LCTEEN_57	Curaray	INIAP-EETP	Ecuador	Loreto, Napo
Theobroma cacao	LCTEEN_63	Curaray	INIAP-EETP	Ecuador	Rio San Miguel, Napo
Theobroma cacao	Tiwi_35	Curaray	INIAP-ECCA	Ecuador	San José de Morona
Theobroma cacao	Tiwi_36	Curaray	INIAP-ECCA	Ecuador	San José de Morona
Theobroma cacao	B_240	Nacional	INIAP-EETP	Ecuador	Pacific coast
Theobroma cacao	PAL13_A1	Nacional	INIAP-EETP	Ecuador	Palanda
Theobroma cacao	PAL15_A1	Nacional	INIAP-EETP	Ecuador	Palanda
Theobroma cacao	PAL35_A5	Nacional	INIAP-EETP	Ecuador	Palanda
Theobroma cacao	SA_16	Nacional	INIAP-EETP	Ecuador	Pacific coast
Theobroma cacao	SNA_1001	Nacional	INIAP-EETP	Ecuador	Pacific coast
Theobroma cacao	SNA_1003	Nacional	INIAP-EETP	Ecuador	Pacific coast
Theobroma cacao	SNA_604	Nacional	INIAP-EETP	Ecuador	Pacific coast
Theobroma cacao	RB_29	Purus	CATIE	Brazil	Rio Branco, Acre
Theobroma cacao	RB_37	Purus	CATIE	Brazil	Rio Acre, Acre
Theobroma cacao	RB_41	Purus	CATIE	Brazil	Rio Branco, Acre
Theobroma cacao	RB_43	Purus	CATIE	Brazil	Rio Branco, Acre
Theobroma cacao	RB_46	Purus	CATIE	Brazil	Rio Branco, Acre
Theobroma cacao	RB_48	Purus	CATIE	Brazil	Rio Branco, Acre
Theobroma cacao	RB_49	Purus	CATIE	Brazil	Rio Branco, Acre
Theobroma cacao	SCA_11	Contamana	Cirad-Montpellier	Peru	Rio Ucayali
Theobroma cacao	SCA_12	Contamana	CRC	Peru	Rio Ucayali
Theobroma cacao	SCA_5	Contamana	CRC	Peru	Rio Ucayali
Theobroma cacao	SCA_6	Contamana	CRC	Peru	Rio Ucayali
Theobroma cacao	PA_126	marañón	CRC	Peru	Parinari-Marañon river
Theobroma cacao	PA_191	Marañón	CRC	Peru	Parinari-Marañon river
Theobroma cacao	PA_293	Marañón	CRC	Peru	Parinari-Marañon river
Theobroma cacao	PA_296	Marañón	CRC	Peru	Parinari-Marañon river
Theobroma cacao	PA-279	Marañón	CRC	Peru	Parinari-Marañon river
Theobroma cacao	PA-299	Marañón	CRC	Peru	Parinari-Marañon river
Theobroma cacao	PA-71	Marañón	CRC	Peru	Parinari-Marañon river
**Species**	**Accession**	**Genetic group**	**Preservation collection**	**Country of origin**	**Place of collection**

Species	Accession	Genetic group	Preservation collection	Country of origin	Place of collection
Theobroma cacao	B_97 61/B2	Criollo	CRC	Belize	Toledo district
Theobroma cacao	B97_EX1_C3	Criollo	CRC	Belize	Toledo district
Theobroma cacao	CACAO_2	Criollo	Cirad-DNA stock	México	Yucatan
Theobroma cacao	COL_10	Criollo	CIC	Colombia	Magdalena
Theobroma cacao	HABILLAL	Criollo	Cirad-DNA stock	México	Michoacan
Theobroma cacao	HE_4	Criollo	Cirad-DNA stock	Venezuela	Tachira
Theobroma cacao	LAN_7	Criollo	Cirad-DNA stock	México	Lacandona forest
Theobroma cacao	LIB_3	Criollo	INTA	NICARAGUA	La libertad de Chontalpa
Theobroma cacao	EBC_10_401	Caqueta	Cirad	Colombia	Rio Caqueta, Amazonas
Theobroma cacao	EBC_29	Caqueta	INIAP-ECCA	Colombia	Rio Caqueta, Amazonas
Theobroma cacao	EBC_30	Caqueta	INIAP-ECCA	Colombia	Rio Caqueta, Amazonas
Theobroma cacao	EBC_48	Caqueta	INIAP-ECCA	Colombia	Rio Caqueta, Amazonas
Theobroma cacao	EBC_5	Caqueta	Cirad	Colombia	Rio Caqueta, Amazonas
Theobroma cacao	EBC_6_401	Caqueta	Cirad	Colombia	Rio Caqueta, Amazonas
Theobroma cacao	EBC_6_S10	Caqueta	INIAP-EETP	Colombia	Rio Caqueta, Amazonas
Theobroma cacao	EBC_9_A2	Caqueta	INIAP-ECCA	Colombia	Rio Caqueta, Amazonas
Theobroma cacao	LCTEEN_218	Curaray	INIAP-EETP	Ecuador	Rio Villano, Para
Theobroma cacao	LCTEEN_227	Curaray	INIAP-EETP	Ecuador	Rio Villano, Para
Theobroma cacao	LCTEEN_312	Curaray	INIAP-EETP	Ecuador	Rio Upano
Theobroma cacao	LCTEEN_333	Curaray	INIAP-EETP	Ecuador	Rio Upano
Theobroma cacao	LCTEEN_57	Curaray	INIAP-EETP	Ecuador	Loreto, Napo
Theobroma cacao	LCTEEN_63	Curaray	INIAP-EETP	Ecuador	Rio San Miguel, Napo
Theobroma cacao	Tiwi_35	Curaray	INIAP-ECCA	Ecuador	San José de Morona
Theobroma cacao	Tiwi_36	Curaray	INIAP-ECCA	Ecuador	San José de Morona
Theobroma cacao	B_240	Nacional	INIAP-EETP	Ecuador	Pacific coast
Theobroma cacao	PAL13_A1	Nacional	INIAP-EETP	Ecuador	Palanda
Theobroma cacao	PAL15_A1	Nacional	INIAP-EETP	Ecuador	Palanda
Theobroma cacao	PAL35_A5	Nacional	INIAP-EETP	Ecuador	Palanda
Theobroma cacao	SA_16	Nacional	INIAP-EETP	Ecuador	Pacific coast
Theobroma cacao	SNA_1001	Nacional	INIAP-EETP	Ecuador	Pacific coast
Theobroma cacao	SNA_1003	Nacional	INIAP-EETP	Ecuador	Pacific coast
Theobroma cacao	SNA_604	Nacional	INIAP-EETP	Ecuador	Pacific coast
