# Supplementary material for: Cacao in the Mississippian World: Archaeogenomic evidence for T. cacao consumption at the Etowah Site, Georgia
Source: PLoS One. 2026 Aug 26;21(8):e0353607. doi: 10.1371/journal.pone.0353607 (PMC13514064; doi:10.1371/journal.pone.0353607)
Supplement: Supplementary file 4 [file pone.0353607.s006.docx]

Primer name	Gene/sequence name	Sequence name	Sequence reference	Forward primer	Tm forw.	Reverse primer	Tm rev.	Product length (bp)
**MITO-6**	Cytochrome C oxydase subunit 2	ESTTik/CL294Contig1	Argout et al. (2008)	AGCAACACCTATGATGCAAGGA	60.03	AGCACGAACCAAGATCCGTA	59.11	**100**
**MITO-290**	Cytochrome C oxydase subunit 2	ESTTik/CL294Contig1	Argout et al. (2008)	CCTTGTGATGCAGCGGAAC	59.50	TCGTCCATTGAGTATAAGAGAGCA	59.11	290
