# Supplementary material for: Cacao in the Mississippian World: Archaeogenomic evidence for T. cacao consumption at the Etowah Site, Georgia
Source: PLoS One. 2026 Aug 26;21(8):e0353607. doi: 10.1371/journal.pone.0353607 (PMC13514064; doi:10.1371/journal.pone.0353607)
Supplement: Supplementary file 2 [file pone.0353607.s003.docx]

>Etowah 2_MX17_494

TCCATGTAATAACCCGTACTACATGCCTACTTTTTCCCAACTCCACCACAACCACCTCCACTTATAACCT

>Etowah 2_MX17_495

TCCATGTAATAACCCGTACCACATGCCTACTTTTTCCCAACTCCACCACAACCACCTCCACTTATAACCT

>Etowah 2_MX17_direct_349

TTGACCAATGCAAGAGCTTTGAAAGAGGCATCATCAAGAGGTGTAAAAATTGTGATAGCCACAGGAAAAGTAAGAACAGAAGGAACTTTTTGTTCTC

>Etowah 2_MX17_direct_1328

TCGTTTAACAGGGCTTTGCTACATGGAGCAGTCATCTCTGCTTTTCTGGCTATTGCTCAAGCC

>Etowah 3_MX11-A1-T2_36

TTGCTTTTCTTTCTTCCTCTCACTTGTTTTCCTTGACGTCTCTGACTAACTACTGAAGGAGGAACACTTGTCCCTGTGAACTTTGTTGTCTTAGGTGACGAAGATTCTGTTTTAACTGCAGTGTCCCTTTTCGGTGCTGATAACAAAGAAAATTCGGGTTGTGATGCAGT

>Etowah 3_MX11-A1-T1_328

AAGGGATTCTACCTCTACAGCTGCTTTGAGCTGCAGCAATTT

>Etowah 3_MX11-A1-T1_258

AAGGAGATTTGCTAAATCAAATCTGCAATTTTCAATGGAAAACCATGAATTACCCACCATGGAAGTCAACATCTG

>Etowah 3_MX11-A1-T1_251

ACTGCAGATGTTGACTTCCATGGTGGGTAATTCATGGTTTTCCATTGAAAATTGCAGATTTGATTTAGCAAATCTCCTT

>Etowah 3_MX11-A1-T1_252

AAGGAGATTTGCTAAATCAAATCTGCAATTTTCAATGGAAAACCATGAATTACCCACCATGGAAGTCAACATCTGCAGT

>Etowah 3_MX11-A2-T4_232

ACTGCAGATGTTGACTTCCATGGTGGGTAATTCATGGTTTTCCATTGAAAATTGCAGATTTGATTTAGCAAATCTCCTT

>Etowah 3_MX11-A2-T4_233

AAGGAGATTTGCTAAATCAAATCTGCAATTTTCAATGGAAAACCATGAATTACCCACCATGGAAGTCAACATCTGCAGT

>Etowah 3_MX11-A1-T1_105

GGACTCATAGGGTGCCAAGGATTAGGTGGAACATTATCCCCCATTGCATCACCAAAGTTGGGTGCTGGTCTGCAGTGGTCAACATGAAAACATAAGTACAGAAAAATAAACCAAATAAAGTAAGGAGACGGCTAAAAT

>Etowah 3_MX11-A1-T1_106

ATTTTAGCCGTCTCCTTACTTTATTTGGTTTATTTTTCTGTACTTATGTTTTCATGTTGACCACTGCAGACCAGCACCCAACTTTGGTGATGCAATGGGGGATAATGTTCCACCTAATCCTTGGCACCCTATGAGTC

>Etowah 3_MX11-A1-T1_120

ATATCCCAAAATGGAGGCTGATGAAAATAATTAATTACAAGAAGTTGCTTTGACCGTAGGTTGGGTAATAGCTGCATTGCACCCATAGCAACTGGTAGAGCATCATCAGAGTCACGTAGTGATGATAAAAAA

>Etowah 3_MX11-A1-T2_102

GTTTTTTATCATCACTACGTGACTCTGATGATGCTCTACCAGTTGCTATGGGTGCAATGCAGCTATTACCCAACCTACGGTCAAAGCAACTTCTTGTAATTAATTATTTTCATCAGCCTCCATTTTGGGATAT

>Etowah 3_MX18_direct_54

TGTCATTTACATGTTAAGAGTTATTTGATTATGATTGTAATTTCTATTTGTATATATAACTTCAAACCAAAATTGCTTGATTAGTTAAGTCAATATCTTCTCATCG

>Etowah 25_MX10-A2-T4_145

GCAGTGTATTGAAAGTACAGATAAAAGTTGAGAAACCATGCCTAAGATCTAAAGAAGCAAAATTCATAACTTAACTCTTTCAGTTTCTTGCGT

>Etowah 25_MX10-A2-T4_140

TACGCAAGAAACTGAAAGAGTTAAGTTATGAATTTTGCTTCTTTAGATCTTTGGCATGGTTTCTCAACTTTTATCTGTACTTTCAATACACTGC

>Etowah 25_MX10-A2-T4_230

TTGCTGCAAAAGTTTATCCTTTCCTGAGGAGTTAATCTGATCAAGCCGCTTCTTCAGAAATGGC

>Etowah 25_MX10-A1-T1_132

AAGGAGATTTGCTAAATCAAATCTGCAATTTTCAATGGAAAACCATGAATTACCCCCCATGGAAGTCAACATCTGC

>Etowah 25_MX10-A1-T1_106

AAGGAGATTTGCTAAATCAAATCTGCAATTTTCAATGGAAAACCATGAATTACCCACCATGGAAGTCAACATCTGCAGT

>Etowah 25_MX10-A1-T1_108

AAGGAAATTTGCTAAATCAAATCTGCAATTTTCAATGGAAAACCATGAATTACCCACCATGGAAGTCAACATCTGCAGT

>Etowah 25_MX10-A1-T1_109

ACTGCAGATGTTGACTTCCATGGTGTGTAATTCATGGTTTTCAATTGAAAATTGCAGATTTGATTTAGCATATCTCCTT

>Etowah 25_MX10-A2-T4_186

AAGGAGATTTGCTAAATCAAATCTGCAATTTTCAATGGAAAACCATGAATTACCCACCATGGAAGTCAACATCTGCAGT

>Etowah 25_MX10-A2-T4_189

AAGGAGATTTGCTAAATCAAATCTGCAATTTTCAATGGAAAAACATGAATTACCCACCATGGAAGTCAACATCTGAAGT

>Etowah 25_MX10-A2-T4_191

ACTGCAGATGTTGACTTCCATGGTGGGTAATTCATGGTTTTCCATTGAAAATTGCAGATTTGATTTAGCAAATCTCCTT

>Etowah 25_MX10-A2-T4_190

ACTGCAGATGTTGACTTCCATGGTGGGTAATTCATGGTTTTCCATTGAAAATTGCAGATTTGATTTAGCAAATCTCCTC

>Etowah 25_MX10-A1-T1_159

GGTTGTTGATTTGGACATGCCTGGCTCATTTGGGGTCGGTTCACTGCAGACTGATGTATACACATG

>Etowah 25_MX10-A2-T4_51

ATTTTAGCCGTCTCCTTACTTTATTTGGTTTATTTTTCTGTACTTATGTTTTCATGTTGACCACTGCAGACCAGCACCCAACTTTGGTGATGCAATGGGGGATAATGTTCCACCTAATCCTTGGCACCCTATGAGTCC

>Etowah 25_MX10-A2-T4_52

ATTTTAGCCGTCTCCTTACTTTATTTGGTTTATTTTTCTGTACTTATGTTTTCATGTTGACCACTGCAGACCAGCACCCAAATTTGGTGATGCAATGGGGGATAATGTTCCACCTAATCCTTGGCACCCTATGAGTCC

>Etowah 25_MX10-A1-T1_43

ATATTACTCTTATGTCCATTCACAGTCACAAAATTACGAAATACTCCTCTACAAAACTTTAAAGGTTGGTTGGAGTGCACTGAAGGGTAAAGCTGGAAGCAAGCAATTGTAAAGGATTTATAGTG

>Etowah 25_MX10-A2-T4_82

ATATTACTCTTATGTCCATTCACAGTCACAAAATTACGAAATACTCCTCTACAAAACTTTAAAGGTTGGTTGGAGTGCACTGAAGGGTAAAGCTGGAAGCAAGCAATTGTAAAGGATTTATAGTG

>Etowah 25_MX10-A1-T1_197

CTCCACCTTCTCCAATTGAAACTCCGAATTGTTCCTCCAAGGCCATCATT

>Etowah 25_MX10-A2-T4_268

CTCCACCTTCTCCAATTGAAACTCCGAATTGTTCCTCCAAGGCCATCATT
